# Intramolecular Hydrogen Bonds in Normal and Sterically Compressed *o*-Hydroxy Aromatic Aldehydes. Isotope Effects on Chemical Shifts and Hydrogen Bond Strength

**DOI:** 10.3390/molecules24244533

**Published:** 2019-12-11

**Authors:** Poul Erik Hansen, Fadhil S. Kamounah, Bahjat A. Saeed, Mark J. MacLachlan, Jens Spanget-Larsen

**Affiliations:** 1 Department of Science and Environment, Roskilde University, Universitetsvej 1, DK-4000 Roskilde, Denmark; spanget@ruc.dk; 2 Department of Chemistry, University of Copenhagen, Universitetsparken 5, DK-2100 Copenhagen, Denmark; fadil@chem.ku.dk; 3 Department of Chemistry, College of Education for Pure Sciences, University of Basrah, Basrah 61004, Iraq; bahjat.saeed@yahoo.com; 4 Department of Chemistry, University of British Columbia, 2036 Main Mall, Vancouver, BC V6T 1Z1, Canada; mmaclach@chem.ubc.ca

**Keywords:** isotope effects on chemical shifts, Steric compression, hydrogen bond strength, MP2 and B3LYP calculations, *o*-hydroxy aromatic aldehydes, atoms-in-molecules

## Abstract

A number of *o*-hydroxy aromatic aldehydes have been synthesized to illustrate the effect of steric compression and O···O distances on the intramolecular hydrogen bond and the hydrogen bond energies. Hydrogen bond energies have been calculated using the ‘hb and out’ method using either the MP2 method or the B3LYP functional with the basis set 6-311++G(d,p). However, several compounds cannot be treated this way. Hydrogen bond energies are also determined using electron densities at bond critical points and these results are in good agreement with the results of the ‘hb and out’ model. Two-bond deuterium isotope effects on ^13^C chemical shifts are suggested as an experimental way to obtain information on hydrogen bond energies as they easily can be measured. Isotope effects on aldehyde proton chemical shifts have also been measured. The former show very good correlation with the hydrogen bond energies and the latter are related to short O···O distances. Short O···O distances can be obtained as the result of short C=C bond lengths, conjugative effects, and steric compression of the aldehyde group. Short O···O distances are in general related to high hydrogen bond energies in these intramolecularly hydrogen-bonded systems of resonance assisted hydrogen bond (RAHB) type.

## 1. Introduction

Hydrogen bonding, hydrogen bond strength and hydrogen bond energy are still subjects that attract much attention both in chemistry and in biology [1,2,3,4,5,6,7,8,9]. Intramolecular hydrogen bonding of the resonance assisted type is important in DNA bases themselves [8], in co-factors such as pyridoxal-5′-phosphatate and the related aldimines [9], and also in other small molecule such as gossypol treated in this study. Intramolecular hydrogen bonding also has influence on uptake over membranes. In this investigation we consider the intramolecular hydrogen bonding in a number of *o*-hydroxy aromatic aldehydes. In a qualitative fashion, the strength can be judged from OH stretching frequencies [10], OH chemical shifts, two-bond deuterium isotope effects on ^13^C chemical shifts [11] or O···O distances. In a more quantitative fashion, the energy difference between the hydrogen-bonded and the open form (OH group turned 180° about the C-O axis) (‘hb and out’) has been suggested as a theoretical measure of the hydrogen bond energy [12]. This method has mainly been used in intermolecular systems but also in a few cases in intramolecularly hydrogen-bonded systems [7,13]. It requires that the OH group of the open form is not involved in steric or other disturbing interactions. Other methods, such as the molecular tailoring approach [14] has recently been studied by Rusinska-Roszak [15]. Hydrogen bond energies have also been estimated from Bader’s atoms–in-molecules (AIM) analysis [16] using electron densities at the bond critical points. Only a few experimental estimates of hydrogen bond energies have been published. Reuben [17] suggested a correlation between hydrogen bond energies and two-bond deuterium isotope effects on ^13^C chemical shifts based on the work of Schaefer [18]. This led to the equation ln(^2^Δ) = 2.783 + 0.34 · E, in which E is the hydrogen bond energy in kcal/mol and the two-bond isotope effect is in ppb. This equation was later used in proteins [19,20].

*o*-Hydroxy aromatic aldehydes are very suitable molecules for investigating hydrogen bond energies estimated by the ‘hb and out’ method as the CH proton has no special interactions in the “out” molecule (see later). Previously, related systems such as *o*-hydroxyacyl aromatics have been studied and showed rather strong hydrogen bonds, partly due to steric compression, but it was also found that steric compression could lead to non-planar hydrogen-bonded systems [21,22].

The compounds explored in this paper cover a broad range of steric interactions and bond lengths (bond orders) of the “double” bond (double in quotation marks as it in most cases refers to a bond in an aromatic system) linking the hydrogen bond donor and acceptor. Therefore, they provide a number of scenarios: (i) steric compression of the hydrogen bond partners; (ii) conjugation of the aldehyde group with substituents other than the *o*-hydroxy group; (iii) variation of the length of the CC bond connecting the hydrogen bond donor and acceptor; and (iv) variation of the O···O distance between the oxygen atoms of the hydrogen bond. Researchers have judged the importance of the O···O distance for the hydrogen bond strength very differently [2,23]. Perrin has shown that no obvious correlation exists for charged systems [2].

**Figure 1:**
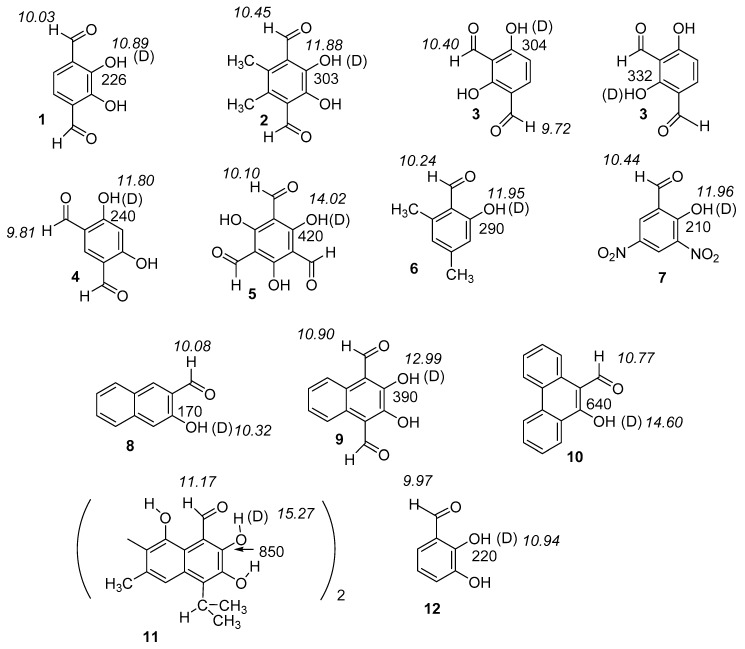
Experimental two-bond deuterium isotope effects in ppb on ^13^C chemical shifts (normal font) and aldehyde and OH proton chemical shifts in ppm (in italics). The structure used in the calculation for **11** is a short version in which the second half is replaced by a phenyl ring. Data for **11** from Ref. 24 (Reproduced with permission from [24]). For a full set of isotope effects on chemical ^13^C chemical shifts see Supplementary Materials Scheme S1.

**Figure 2:**
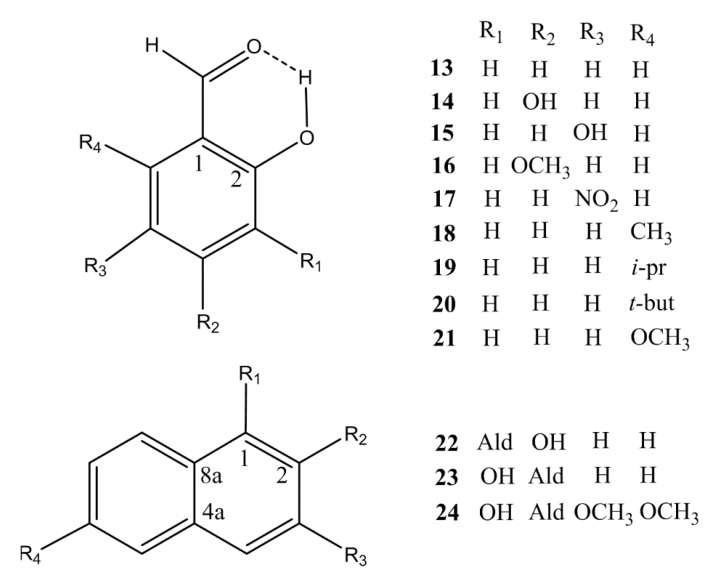
Other compounds investigated. Isotope effects for compounds **13**–**23** can be found in [13] (Reproduced with permission from [13]).

Here we present the results (structures and hydrogen bond energies) of an experimental and a theoretical study of intramolecular hydrogen bonding for a series of non-charged systems. It should be noted that all bond lengths and distances are calculated. Figure 1; Figure 2 show the compounds explored. The compounds in Figure 1 have been selected to have more steric strain and lower or higher π-bond order of the CC bond linking the donor and acceptor group than in most systems previously investigated. The ‘hb and out’ method was chosen for molecules with no disturbing interactions taking place in the open form. The MP2 method was chosen as this had shown good results previously [7]. Electron densities at the bond critical points [16] have also been related to two-bond deuterium isotope effects on ^13^C-NMR chemical shifts.

## 2. Results

### 2.1. Structures and Energies

Structures were optimized and energies calculated in two different ways: MP2 with the basis set 6-311++G(d,p), or using the B3LYP functional with the same basis set. The structure of gossypol (**11**) is extraordinary with a very short O···O distance (2.46 Å) [25]. In 5-nitrosalicylaldehyde (**17**), the nitro group is twisted slightly out of the benzene ring plane in the MP2 calculations, whereas it is in the ring plane in the B3LYP calculations.

**Figure 3:**
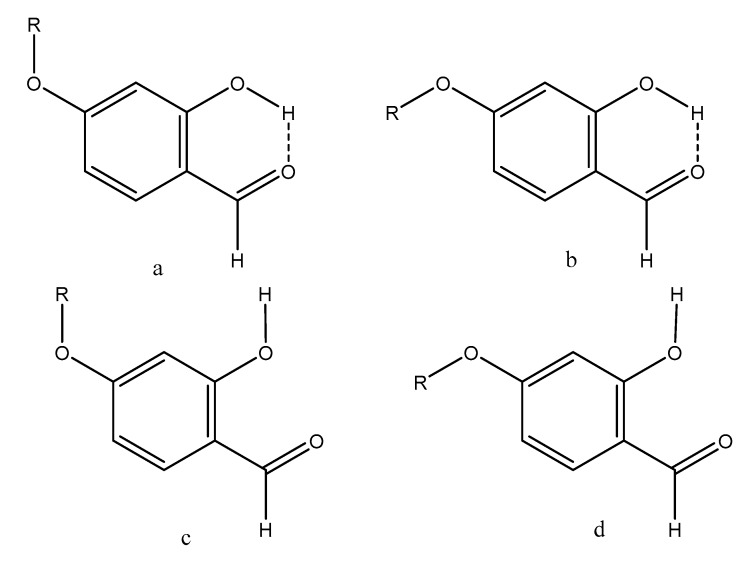
Conformers of **14**–**16**.

The hydrogen-bond energies were estimated by subtracting the computed energies of the non-hydrogen-bonded forms (OH group turned 180°) from the energies of the hydrogen-bonded forms as originally demonstrated for this type of molecules by Cuma, Scheiner, and Kar [12]. Unless otherwise indicated hydrogen bond energies quoted in the following were computed by this ‘hb and out’ method. The hydrogen bond energy estimate was computed only for compounds with no disturbing interference in the rotated forms, meaning that compounds like **1**, **2**, **5**, **7**, **9**, **11**, and **12** are not considered. **3** is also excluded as the C=O group turns out of the plane in the MP2 calculations in the open form, and **7** is excluded because the structure did not converge in the MP2 calculations. However, a number of other compounds are included, such as 6-methylsalicylaldehyde (**18**), 4- and 5-hydroxysalicylaldehyde (**14**) and (**15**), 4-methoxysalicylaldehyde (**16**) (see Figure 3) and 5-nitrosalicylaldehyde (**17**), 2-hydroxynaphthaldehyde (**22**) and 3,6-dimethoxy-1-hydroxy-2-naphthaldehyde (**24**) (see Figure 2). 6-isopropylsalicylaldehyde (**19**) and 6-*t*-butylsalicylaldehyde (**20**) have also been calculated, although the compounds have not been synthesized.

A possible complication in the application of the ‘hb and out’ method as discussed above is interactions in the open form. The OH bond lengths of the open forms of the calculated benzene derivatives are similar for all compounds indicating that no unusual interactions take place. As seen in Figure S2, a good correlation is observed between the ‘hb and out’ hydrogen bond energies calculated by the MP2 method and by the B3LYP functional. In this paper, we mostly show plots with MP2 values, but the trends are the same with B3LYP values.

**Figure 4:**
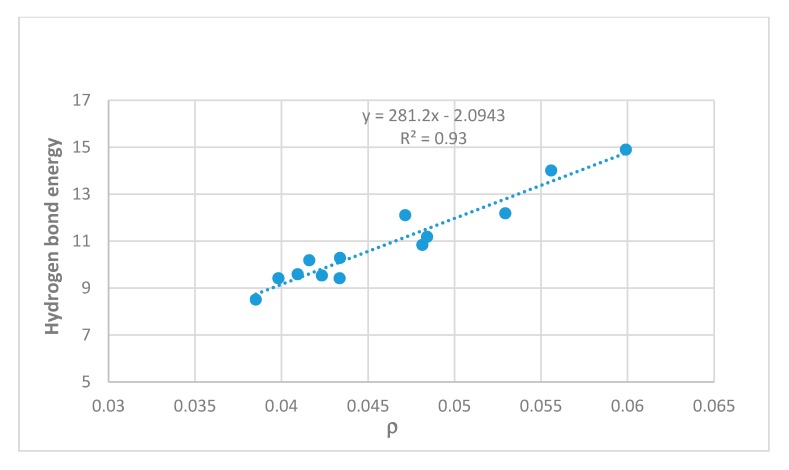
Hydrogen bond energies (MP2, ‘hb and out’) in kcal/mol vs. electron density at the bond critical points (ρ) in atomic units. Compounds investigated are **5**, **7**, **8**, **10**, **12**–**15**, **17**, **21,** 5-chlorosalicylaldehyde, and 5-methyl-1,3-benzenedialdehyde.

**Figure 5:**
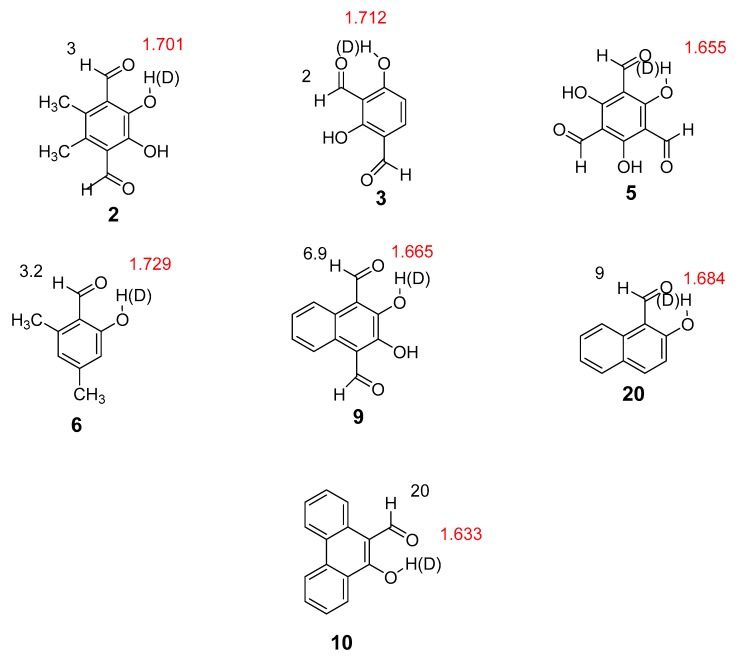
Experimental deuterium isotope effects on aldehyde proton ^1^H-NMR chemical shifts (ppb, in black) and OH···O=C calculated distances (Å, in red).

Energies can also be estimated on the basis of electron densities at the bond critical points, as discussed for example by Afonin et al. [5]. A good correlation is found with ‘hb and out’ calculated energies as seen in Figure 4. Steric compression clearly influences the OH···O=C distances as seen in Figure 5 and energies as demonstrated later.

For the compounds 4-hydroxy- (**14**), 4-methoxy- (**16**) and 5-hydroxysalicylaldehyde (**15**), the ‘hb and out’ hydrogen bond energies depended on the orientation of the substituent in 4- or 5-position as discussed in the following. The a-form of Figure 3 has the lowest energy for the hydrogen bonded ones and the d-form for the open forms. The characteristics of the two forms are that in these forms the OH- and OR-bonds are more or less perpendicular. Hence, the calculated ‘hb and out’ hydrogen bond energies depend significantly on the orientation of the substituent, and for 4-methoxysalicylaldehyde (**16**) the difference is as large as 1.4 kcal/mol.

For **4** the “doubly” open form has also been calculated. In this case the calculated energy is 19.9 kcal/mol compared to the value of 9.4 kcal/mol for one OH group turned.

### 2.2. Deuterium Isotope Effects on Chemical Shifts

#### 2.2.1. Deuterium Isotope Effects on ^13^C Chemical Shifts

Deuterium isotope effects are defined as *^n^*ΔC = δC(OH)-δC(OD), *n* being the number of bonds between the deuterium and the atom in question and exemplified with ^13^C-NMR. Data for salicylaldehydes (**13**–**17**) and hydroxynaphthaldehydes (**22**–**24**) are taken from [17,21]. Both types of data have been previously used in the discussion of hydrogen bond strength. The discussion is centered around two-bond deuterium isotope effects on ^13^C chemical shifts (TBDIE). A comparison of 4,6-dimethylsalicyaldehyde- and salicylaldehyde shows larger two-bond isotope effects in the former. In a similar way, for 5,6-dimethyl-2,3-dihydroxy-1,4-benzenedialdehyde and 2,3-dihydroxy-1,4-benzenedialdehyde, the former shows larger two-bond isotope effects. Gossypol (**11**) shows a very large TBDIE of 0.85 ppm [22]. This value is much larger than found in 2-hydroxynaphtaldehyde (**21**) (0.411 ppm) [21] or in **9** (Figure 2). Another interesting observation is the almost identical two-bond isotope effects of 5-nitrosalicylaldehyde (**17**), 3,5-dinitrosalicylaldehyde (**7**), and salicylaldehyde (**13**).

**Figure 6:**
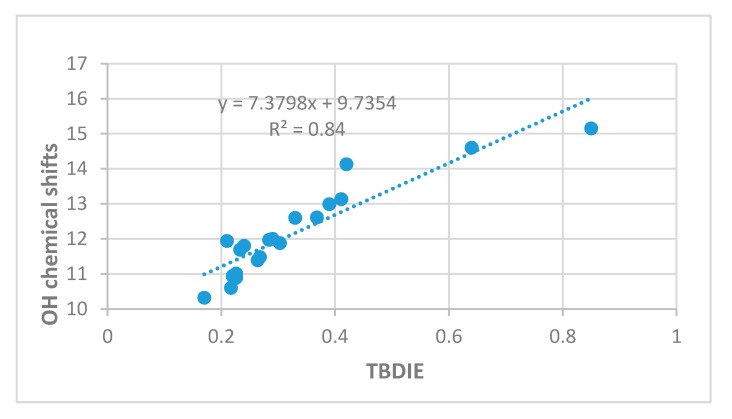
OH chemical shifts in ppm vs. two-bond deuterium isotope effects on ^13^C chemical shifts (TBDIE) in ppm.

OH chemical shifts have been used to gauge hydrogen bond strength. As seen in Figure 6 a reasonable correlation is found between OH chemical shifts and TBDIE.

#### 2.2.2. Deuterium Isotope Effects on ^1^H Chemical Shifts

As seen from Figure 5, deuterium isotope effects on aldehyde proton chemical shifts can be found in some of the compounds. Although these are small, because the chemical shift range of ^1^H is small, they easily can be measured. Isotope effects on aldehyde proton chemical shifts were observed in all compounds with OH···O=C distances shorter than 1.729 Å (Figure 5).

### 2.3. Correlations

**Figure 7:**
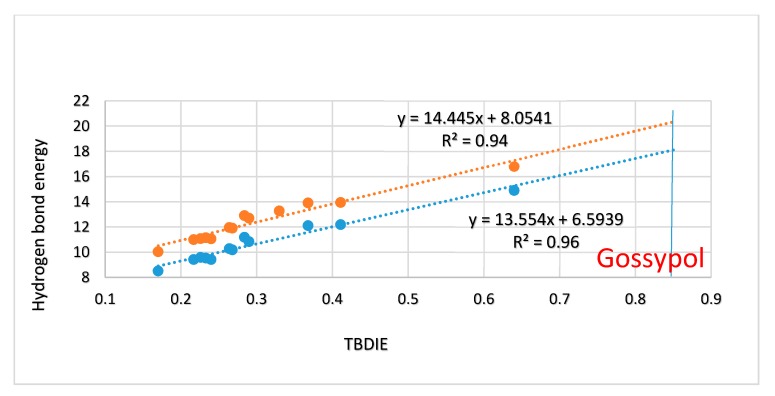
Plot of ‘hb and out’ hydrogen bond energies in kcal/mol calculated either with MP2/6-311++G(d,p) or with B3LYP/6-311++G(d,p) vs. observed two-bond deuterium isotope effect (TBDIE) on ^13^C chemical shifts in ppm. Top correlation line is B3LYP, bottom one is MP2. The observed TBDIE for gossypol is marked by the vertical blue line.

In Figure 4 energies estimated from the ‘hb and out’ method (MP2) are plotted vs. electron densities at the bond critical points (ρ). A rather satifactory linear correlation is found; the plot using B3LYP results is very similar (not shown). A plot of the ‘hb and out’ energies calculated with the MP2 method and with the B3LYP functional vs. observed two-bond deuterium isotope effect (TBDIE) on ^13^C chemical shifts is shown in Figure 7. Again, good correlations are observed.

**Figure 8:**
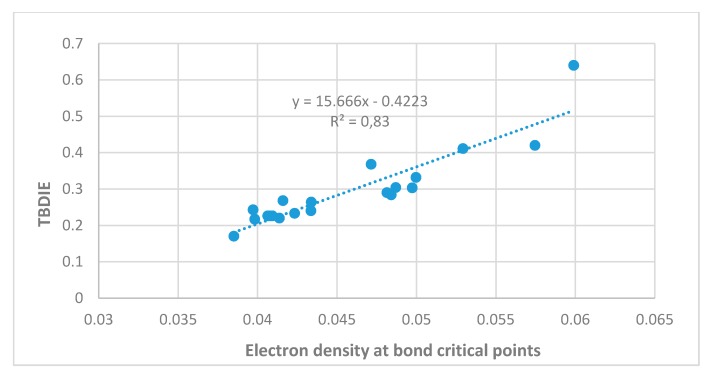
Experimental TBDIE’s vs. electron densities at bond critical points.

Two-bond deuterium isotope effects on ^13^C-NMR chemical shifts (TBDIE) are claimed to be good measures for hydrogen bond strength [11]. This claim is supported by the results shown in Figure 7. In Figure 8 TBDIE’s are plotted vs. electron densities at the bond critical point. A much larger array of compounds can then be included. It is seen, that especially data for **5** and **10** are falling off the line.

**Figure 9:**
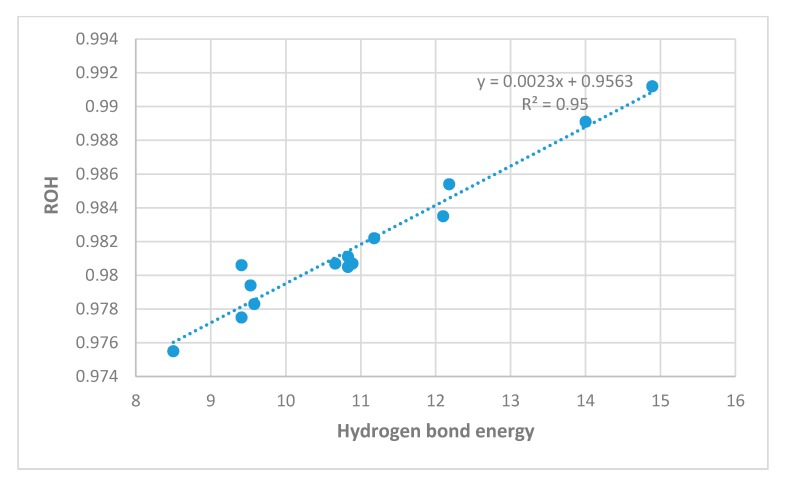
Calculated OH bond lengths in Å vs. ‘hb and out’ hydrogen bond energies in kcal/mol (MP2).

**Figure 10:**
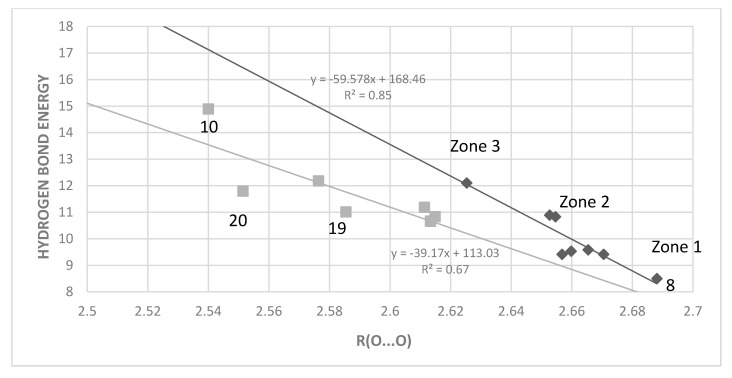
Hydrogen bond ‘hb and out’ energies (kcal/mol) vs. O···O distances (Å) computed with MP2. The data are divided into non-sterically hindered compounds (black diamonds, upper correlation line) with compounds like **4**, **8**, **13**–**17** and sterically hindered ones (grey squares, lower correlation line).

In Figure 9, OH bond lengths show excellent correlation with hydrogen bond energies. The main outlier is **4**, a compound with two hydrogen-bonded systems. A plot of the hydrogen bond energies vs. the O···O distances is given in Figure 10. The correlations are reasonable.

The plot of Figure 10 shows that in general a shorter the O···O distance is related to a higher hydrogen bond energy, but also that it is necessary to divide the compounds into two groups, sterically hindered and non-sterically hindered. The two lower outliers among the sterically hindered ones are 6-*t*-butyl- (**20**) and 6-isopropylsalicylaldehyde (**19**), whereas the one above is the phenanthrene derivative (**10**).

**Figure 11:**
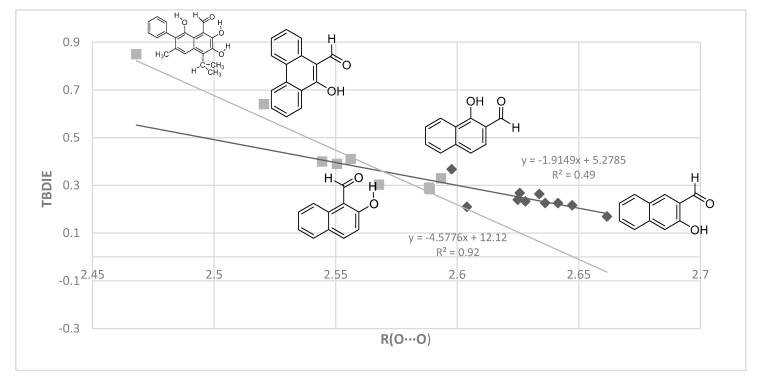
Plot of observed TBDIE vs. O···O distances (Å) computed with MP2. As in Figure 10, black diamonds refer to non-sterically hindered, grey squares to sterically hindered cases. The former include compounds like **4** and **8**, as well as **13**–**17**.

Finally, a plot of experimental TBDIE vs. calculated O···O distances is shown in Figure 11. This allows inclusion of compounds like **1**, **2**, **5**, **7**, **9**, **11** and **12**. The corresponding hydrogen bond energies in the plots in Figure 6, Figure 7, Figure 8 and Figure 9 and 11 are for Boltzmann averaged values, whereas the structural parameters are for the a-form.

## 3. Discussion

One of the aims of the present paper is to relate deuterium isotope effects on chemical shifts to hydrogen bond strength and to describe some of the parameters contributing to hydrogen bond strength in intramolecularly hydrogen-bonded systems. No experimental hydrogen bond energies are available for intramolecularly hydrogen-bonded systems of the kind studied here. In the present study, the hydrogen bond strength is estimated using: (i) the hydrogen bond minus the OH out method (‘hb and out’) [12] and (ii) electron density at the bond critical point. The compounds included in the former type of calculations are only those with no special interactions of the OH group when this is turned 180° around the C-O axis. The main question is whether this method is accurate. In the case of **4** both the conformer with one group turned out and the one with two groups turned out have been studied. As seen in the Results section, the energies are not strictly additive; but it must in this context be remembered, that different orientation of substituents could lead to a variation. It was found (Figure S2) that the MP2/6-311++G(d,p) and the B3LYP/6-311++G(d,p) procedures gave linearly related results, but the B3LYP energies are approximately 1.4 kcal/mol higher (Figure 7).

**Figure 12:**
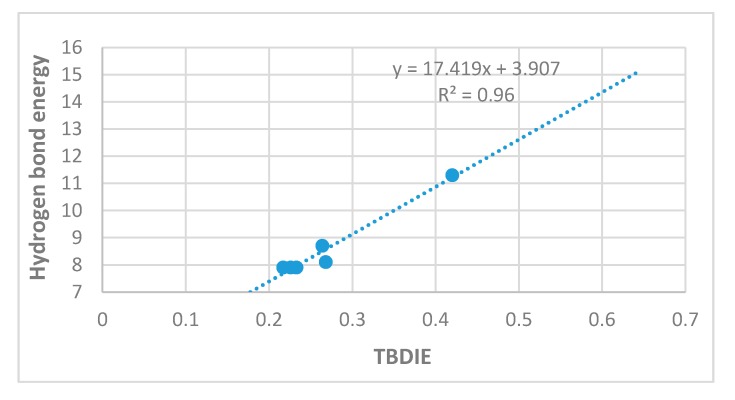
Plot of hydrogen bond energies in kcal/mol calculated by the molecular tailoring approach (MTA) vs. observed TBDIE in ppb. Energies from [15] (Reproduced with permission from [15]).

Rusinska-Roszak [15] has used the molecular tailoring approach (MTA) for a series of *o*-hydroxy aromatic aldehydes, but mostly in a narrow range of hydrogen bond energies as seen in Figure 12. Rusinska-Roszak did not include PAH systems in the MTA analysis; the reason is stated as “polycyclic compounds are not included due to their dependence on the position of the -OH and C=O substituents”. Translated this could mean “because of different degrees of conjugation”. But this is true for most of the compounds investigated by Rusinska-Roszak and certainly affects compound **5** (the point at 0.43 ppm in Figure 12). The molecular tailoring approach method gave mostly smaller hydrogen bond energies than MP2 and B3LYP, but the trends are clearly the same for all three types of calculations (MP2, B3LYP and MTA). The range of energies in our investigation is ~4 kcal/mol. Despite the variation for some compounds due to the orientation of substituents it can be seen that in general, a shorter O···O distance is related to a higher hydrogen bond energy (Figure 10).

**Figure 13:**
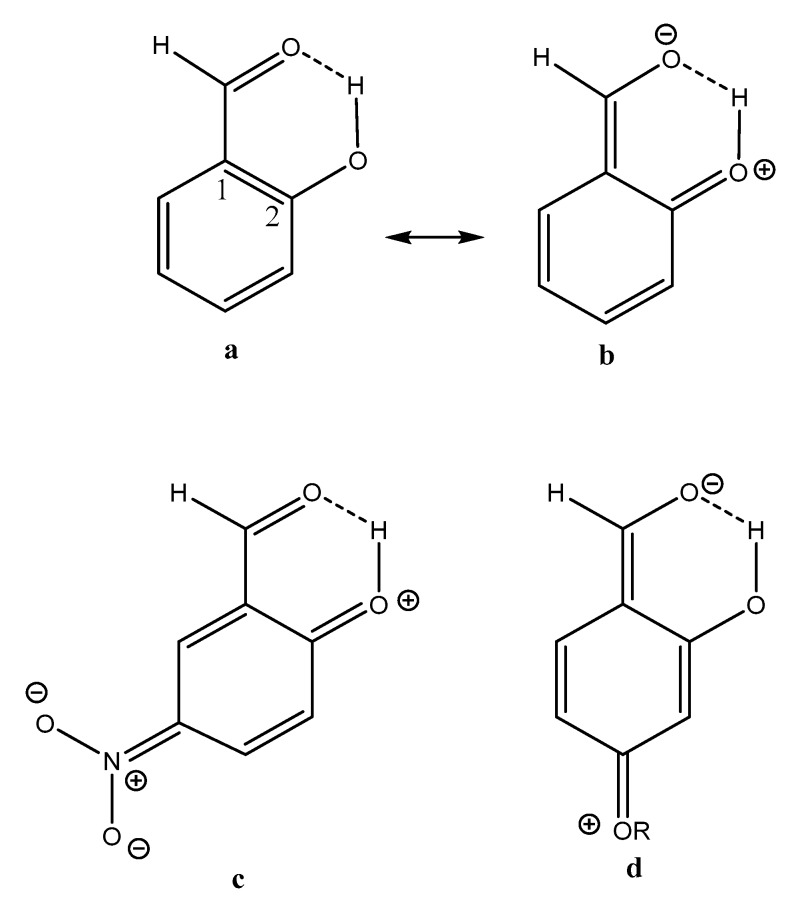
Resonance structures of salicylaldehyde, 5-nitrosalicyladehyde and 4-alkoxysalicylaldehydes.

From the plot if Figure 7 it can also be seen that two-bond deuterium isotope effects (TBDIE) on ^13^C chemical shifts can be used to predict hydrogen bond strengths and the same is true for OH chemical shifts (Figure S4). Obviously, these two parameters show a decent correlation (Figure S3). Two-bond deuterium isotope effects on ^13^C chemical shifts depend to a large extent on the sum of bond orders between the OD deuterium and the carbon in question. The sums of bond orders will in the present compounds increase if the importance of the resonance form c of Figure 13 increases. A short C2-O distance (for an example see Figure 13) will be favored by a short C1-C2 distance due to increased RAHB, but also by a short O···O distance as coulomb interactions will be maximized. Therefore, it is expected, as seen in Figure 7, that the TBDIE is a reasonable measure of hydrogen bond strength.

The observation of deuterium isotope effects on the aldehyde ^1^H resonance (see Figure 5) in compounds with short OH···O=C distances is indicative of orbital overlap. This overlap will likewise strengthen the hydrogen bond.

Factors relevant to intramolecular hydrogen bonding are the interaction between the donor and the acceptor referred to as RAHB, the resonance from electron withdrawing or electron donating substituents [26], coulomb interactions, and orbital overlap. In this respect the O···O distance is an important factor. The significance of the O···O distance has been discussed intensely; some think it is very important [23], others that it is not a good measuring stick for hydrogen bond strength [2].

To a large extent, the O···O distance is determined by the length of the “double” bond connecting the donor and acceptor. In the corresponding hydrocarbons we find the following bond lengths: benzene 1.40 Å, C1-C2 bond of naphthalene 1.36 Å, C2-C3 bond of naphthalene 1.42, and C9-C10 of phenanthrene 1.34 Å. We have divided the data into two groups: non-steric (such as 3-hydroxy-2-naphthaldehyde, salicylaldehyde, 4- and 5-substituted salicylaldehydes and 2,3-dihydroxy-1,4-benzenedialdehyde and 1-hydroxy-2-naphtaldehyde) and steric (4,6-dimethylsalicylaldehyde, 6-methoxysalicylaldehyde, 2-hydroxy-1-naphtaldehyde and 10-hydroxy-9-phenanthrenealdehyde). The trend from Figure 10 is rather clear: a short O···O distance is generally found in compounds with large hydrogen bond energy. Leading to a short distance is steric pressure as in **2**, **3**, **5**, **6**, **9**, and **10**, as well as in **21**. However, it can be described in more detail. In the plot of Figure 10 three “zones” can be recognized for the non-steric ones: compound **8** (first zone), followed by the benzene derivatives (zone 2), and at higher energies the naphthalene derivative 1-hydroxy-2-naphthalene (zone 3). The sterically hindered benzene derivatives are shifted to an O···O distance of ~2.61Å, the sterically hindered naphthalenes to ~2.56 Å, the phenanthrene (**10**) to ~2.52 Å and finally, at the highest energy, is found **11**. The bond length also correlates with the π-bond order of the bond connecting the hydrogen bond donor and acceptor and therefore with the resonance assistance.

The hydrogen bond energy of **6** is 1.25 kcal/mol higher than that of salicylaldehyde. The hydrogen bond energy of **6** is similar to that of 6-methylsalicylaldehyde. For 6-isopropyl-salicylaldehyde the energy is only 0.2 kcal/mol higher than for **6**, but in the case of 6-*t*-butyl-salicylaldehyde (**20**) the energy is 1 kcal/mol higher than for **6**, and the O···O distance is 0.05 Å shorter. Another example is gossypol (**11**). This compound has a very large TBDIE of 0.85 ppm (Figure 11). It has also been reasoned that the compound does not show tautomeric behavior despite the very short O···O distance calculated as 2.47 Å (B3LYP) compared to the X-ray distance of 2.46 Å [25]. From the plot of Figure 7, a hydrogen bond energy of 18-20 kcal/mol can be predicted. It is obvious that part of the reason for the short O···O distance is the short C1-C2 bond, but in the similar naphthalene derivative 2-hydroxynaphtaldehyde the O···O distance is 2.597 Å. Can the extra substituents be responsible for the short distance? In 2,3-dihydroxybenzaldehyde (**12**) the O···O distance is actually longer than in salicylaldehyde. The hydroxyl group in position 8 cannot contribute mesomerically. Gossypol is correctly a ‘dimer’. This is in the present situation mimicked by a phenyl group in position 7. This phenyl group is twisted very heavily out of the naphthalene ring plane (76°) and does not contribute mesomerically, so the short O···O distance has to come from steric compression of the OH group in position 8. Again it is seen that steric compression leads both to a shorter O···O distance and to a higher hydrogen bond energy, but the relationship only holds on a coarse scale.

## 4. Materials and Methods

### 4.1. General Information

3,5-Dimethylphenol, 9-bromophenanthrene, 2-methoxynaphthalene, 3,5-dinitrosalicylaldehyde, and anhydrous magnesium chloride were purchased from TCI Research Chemicals, Haven, Belgium. All other reagents and solvents were analytical grades purchased from Sigma-Aldrich Chemical Co., and used as received unless otherwise stated. Fluka silica gel/TLC-cards 60,778 with fluorescence indicator 254 nm were used for TLC chromatography. Merck silica gel 60 (0.040–0.063 mm, Merck, Darmstadt, Germany was used for flash chromatography purification of the products. ^1^H-NMR and ^13^C-NMR spectra were recorded at 500 MHz and 126 MHz or 400 MHz and 100 MHz on an Ultrashield Plus 500 or 400 spectrometer, Bruker, Fallaenden, Germany using CDCl_3_ or DMSO-*d_6_* as a solvent and TMS as internal standard. LC/MS (ESI) was carried out on a Bruker MicrOTOF-QIII-system with ESI-source with nebulizer 1.2 bar, dry gas 10.0 L/min, dry temperature 220 °C, capillary 4500 V, end plate offset −500 V, Bruker, Hamburg, Germany.

### 4.2. Synthesis

The synthesis of the compounds 2,3-dihydroxy-1,4-benzenedicarboxaldehyde (**1**) [27], 2,3-dihydroxy-5,6-dimethyl-1,4-benzenedicarboxaldehyde (**2**) [28], 2,4-dihydroxy-1,3-benzene-dicarboxaldehyde (**3**) [29], 4,6-dihydroxy-1,3-benzenedicarboxaldehyde (**4**) [29], 2,4,6-trihydroxy-1,3,5-benzenetricarboxaldehyde (**5**) [29], and 2,3-dihydroxy-1,4-naphthalenedicarboxaldehyde (**9**) [28] are described previously.

*4,6-Dimethylsalicylaldehyde* (**6**): Compound **6** was prepared according to the procedure described by Knight et al. [30]. A solution of 3,5-dimethylphenol (1.22 g, 10 mmol) in dry acetonitrile (50 mL) was treated with dry triethylamine (5.22 mL, 37.5 mmol) and anhydrous magnesium chloride (1.43 g, 15 mmol) under nitrogen atmosphere. The mixture was stirred for 15 min. Dry paraformaldehyde (2.03 g, 6.75 mmol) was added under nitrogen atmosphere and the mixture refluxed for 3 h. The solution cooled to room temperature and poured into a cold 5% aqueous HCl solution and left stirred for 45 min. This was extracted with diethyl ether (4 × 40 mL), the ether washed with brine (2 × 30 mL) and dried over anhydrous magnesium sulphate. The solvent was evaporated under reduced pressure and the crude product was purified by flash column chromatography on silica gel using dichloromethane/*n*-hexane (4:1) as eluent, affording the pure product as white crystals (1.00 g, 66%). The NMR data are consistent with those reported in literature [22]. HRMS-ESI calculated for C_9_H_10_O_2_ [M + H]^+^ 150.0680, found 150.0658.

*3-Hydroxy-2-naphthaldehyde* (**8**): The starting material *3-methoxy-2-naphthaldehyde* was prepared according to the procedure described by Legouin et al. [31]. The crude product was purified by flash column chromatography on silica gel using dichloromethane to afford pure product as a pale yellow solid (0.59 g, 56%). The NMR data are consistent with that reported in literature [31]. HRMS-ESI calculated for C_12_H_10_O_2_ [M + H]^+^ 186.0681, found 186.0645. Next a flame-dried 50 mL round-bottom flask equipped with a magnetic stirrer, nitrogen inlet was charged with a solution of 3-methoxy-2-naphthaldehyde (0.21 g, 1.5 mmol) in dry dichloromethane (20 mL) and cooled to 0 °C. BBr_3_ (6.0 mL, 1.0 M solution in dichloromethane) was added under a nitrogen atmosphere and was stirred at 0 °C for 15 min, and at room temperature for 20 h. The reaction mixture was poured into ice and 1.0 M HCl (10 mL). The mixture was stirred for 20 min and extracted with dichloromethane (2 × 30 mL), dried over anhydrous magnesium sulphate and evaporation of solvent under reduced pressure gave a crude product purified by flash column chromatography on silica gel, using dichloromethane to affords pure product as a pale yellow solid (0.14 g, 73%). The NMR data are consistent with that reported in literature except that the two signals at 10.32 ppm must be assigned as the OH resonance and that at 10.08 ppm as the resonance of the aldehyde proton. HRMS-ESI calculated for C_11_H_8_O_2_ [M + H]^+^ 172.0524, found 172.0538.

*9-Methoxyphenanthrene*: This compound was synthesized according to previously reported procedure [31]. The light amber solid was purified by flash column chromatography on silica gel, using dichloromethane/*n*-hexane (2:1) to afford the pure product as off white solid (1.2 g, 91%). The NMR data are consistent with that reported in the literature [31]. HRMS-ESI calculated for C_15_H_12_O [M + H]^+^ 208.0888, found 208.0858.

*10-Methoxy-9-phenanthrenealdehyde*: This compound was synthesized by modification of literature methods [31]. The resulting light brown solid residue was purified by flash column chromatography on silica gel, using an eluent gradient of n-hexane/dichloromethane/(2:1), then (1:1) and finally (1:4) to give a white solid (0.83 g, 67%). The NMR data are in agreement with those of Ref. 31. HRMS-ESI calculated for C_16_H_12_O_2_ [M + H]^+^ 236.0837, found 236.0816.

*10-Hydroxy-9-phenanthrenealdehyde* (**10**): This compound was synthesized by modification of the procedure of [31]. A flame dried 50 mL round-bottom flask equipped with a magnetic stirrer and a nitrogen inlet was charged with a solution of 10-methoxy-9-phenanthrenecarboxaldehyde (0.61 g, 2.6 mmol) in dry dichloromethane (25 mL) and cooled to −10 °C. Under nitrogen atmosphere was added a solution of BBr_3_ (6.0 mL, 1.0 M solution in dichloromethane) and stirred at 0 °C for 15 min and at room temperature for 1.5 h. The reaction mixture was poured onto ice in 1.0 M HCl (30 mL). The mixture was stirred for 30 min and extracted with dichloromethane (2 × 50 mL), dried over anhydrous magnesium sulphate and evaporation of solvent under reduced pressure gave a crude product purified by flash column chromatography on silica gel, using dichloromethane/*n*-hexane (2:1) as eluent to afford the pure product as a pale yellow solid (0.54 g, 96%). The NMR results are similar to those reported [31]. HRMS-ESI calculated for C_15_H_10_O_2_ [M + H]^+^ 222.0681, found 222.0652.

### 4.3. Deuteration

The compounds are deuterated in CDCl_3_ by stirring the dissolved compound with a mixture of D_2_O:H_2_O. After stirring over night the water phase is removed and the CDCl_3_ phase is dried with anhydrous sodium sulfate.

### 4.4. Calculations

Quantum chemical calculations were performed using the Gaussian09 software package (Gaussian.com, Wallingford, CT, USA) [32]. Gas phase equilibrium geometries and model hydrogen bond energies of the investigated molecules were computed with either B3LYP [33,34] density functional theory or MP2 Møller-Plesset second order perturbation theory [35,36] using the 6-311++G(d,p) basis set [37,38,39] and default options [32]. Calculated vibrational frequencies were checked for imaginary values. For a number of species, the geometry optimization failed to converge with MP2/6-311++G(d,p) in spite of several attempts, a problem apparently associated with the inclusion of diffuse functions in the basis set (no problems were observed with MP2/6-311G(d,p)). Theoretical estimates of the intramolecular hydrogen bond energies were obtained by the ‘hb and out’ model: For each species, two geometry optimizations were performed, one for the hydrogen-bonded form, and one for the open form where the OH group is rotated by 180° around the C-O axis. The energy difference between the two forms is taken as an estimate of the energy of the hydrogen bond. The AIM analysis was based on B3LYP/6-311++G(d,p) calculations using the AIMALL17.11 version (AIMAll, Overland Park, KS, USA) [40].

## 5. Conclusions

It is seen that two-bond isotope effects on ^13^C chemical shifts are a good measure of hydrogen bond strength for this type of system, whether the hydrogen bond energy is calculated by the ‘hb and out’ method or estimated from electron densities at bond critical points. Two-bond deuterium isotope effects on ^13^C chemical shifts is an experimental parameter that can easily be measured with very few exceptions. In general, strong hydrogen bonds tend to have short O···O distances. The O···O distance can be modified either by shortening the bond connecting the hydrogen bond donor and the acceptor, by conjugation, or by steric compression of the aldehyde group. Compression of the OH group has no significant effect. Judging from Figure 10 and Figure 11, steric compression seems slightly less effective than short “double” bonds and conjugation.

## Supplementary Materials

The following are available online, Scheme S1: Deuterium isotope effects on ^13^C chemical shifts. Figure S2: Plot of hydrogen bond energies, B3LYP vs. MP; Figure S3: Observed OH chemical shifts in ppm vs. hydrogen bond energies; Table S1: Calculated hydrogen bond energies hb and out method.
