# Supplementary material for: Intramolecular Hydrogen Bonds in Normal and Sterically Compressed o-Hydroxy Aromatic Aldehydes. Isotope Effects on Chemical Shifts and Hydrogen Bond Strength
Source: Molecules. 2019 Dec 11;24(24):4533. doi: 10.3390/molecules24244533 (PMC6943505; doi:10.3390/molecules24244533)
Supplement: Supplementary file 1 [file molecules-24-04533-s001.pdf]

Intramolecular Hydrogen Bonds in Normal and Sterically Compressed *o*-Hydroxy Aromatic Aldehydes. Isotope Effects on chemical shifts and Hydrogen Bond Strength.

Poul Erik Hansen [1], Fadhil S. Kamounah [1,2], Mark J. MacLachlan[3] Bahjat A. Saeed [4] and Jens Spanget-Larsen[1]

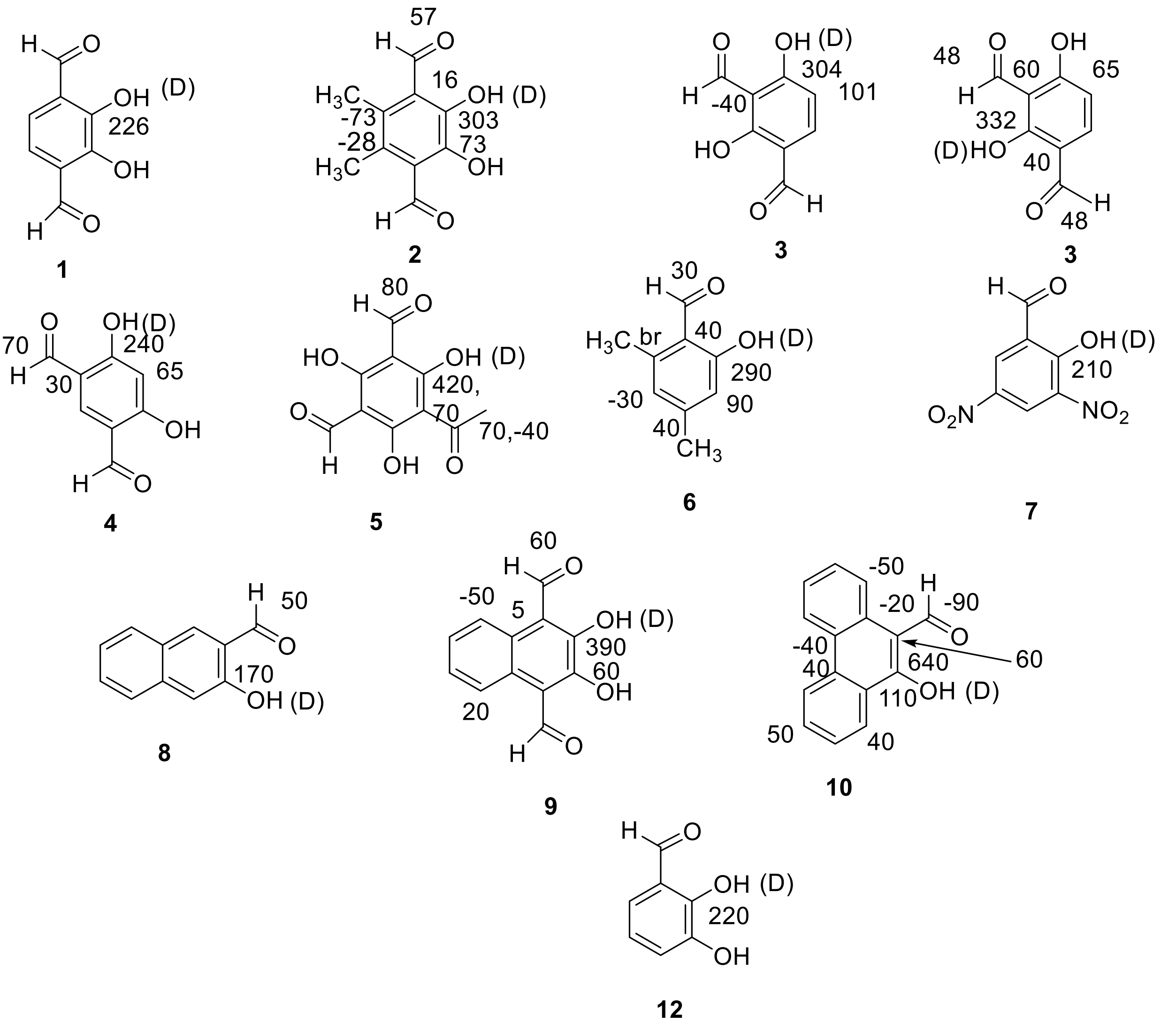

Scheme S1. Deuterium isotope effects on ^{13}C chemical shifts. Carbons not having a number attached did not show an isotope effect. For **5** the second set of isotope effects are due to deuteriation at the other OH group.

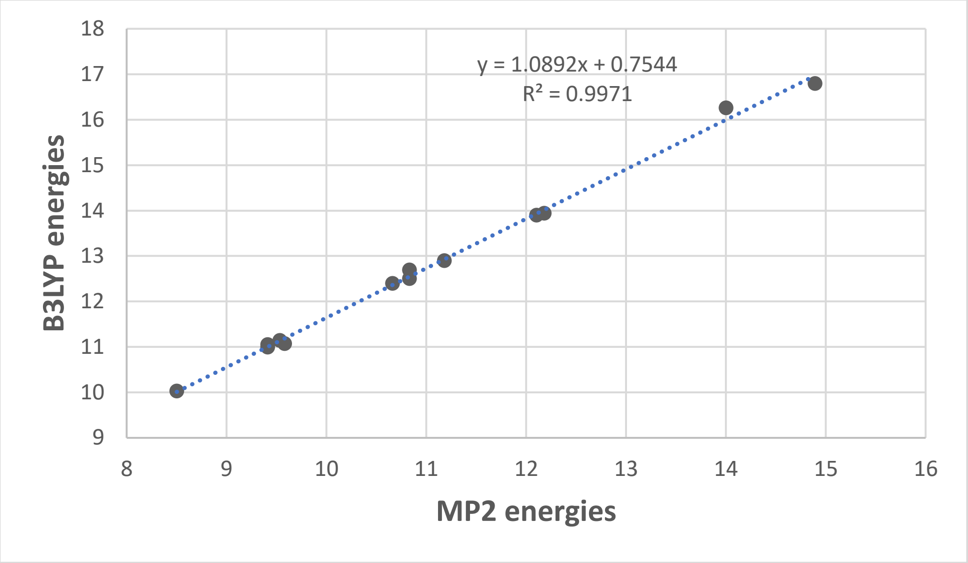

Figure S2. Plot of hydrogen bond energies in Kcal/mole, B3LYP vs. MP2

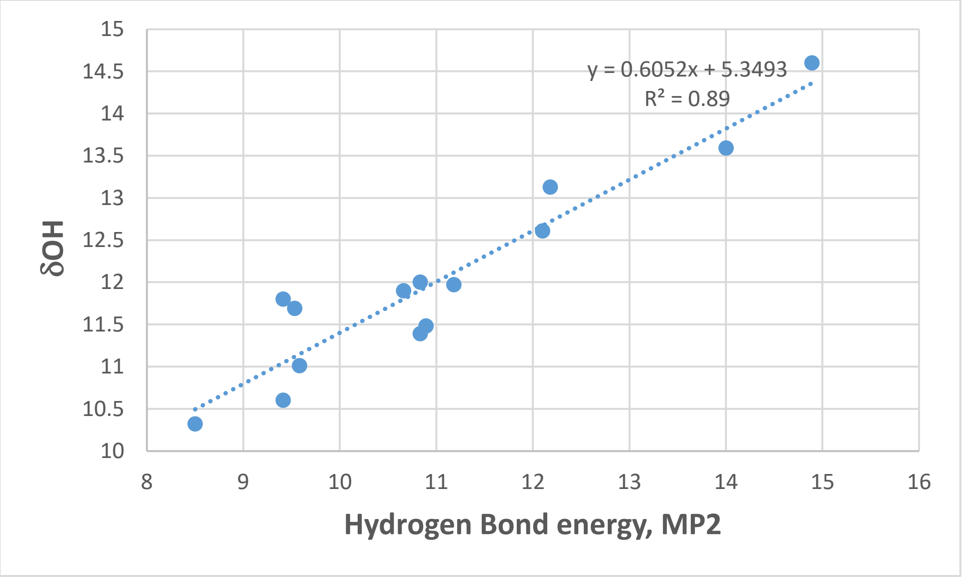

Fig. S3. Observed OH chemical shifts in ppm vs. hydrogen **bond** energies in Kcal/mole (MP2). Data for **13-17** and **21** and **22** are from Ref. 1. The one for 1-hydroxy-3,6-dimethoxy-2-naphthaldehyde (**23**) is from Ref. 2.

Table 1S . Calculated hydrogen bond energies hb and out method. Energies in kcal/mole

Compound	Calc. H-bond energy MP2	Calc. H-bond energy B3LYP
**4**	9.41	11.05
**6**	10.83	12.69
**8**	8.5	10.03
**10**	14.89	16.8
**13**	9.58	11.07
**14**	10.28	11.95
**15**	9.5	10.99
**16**	10.18	11.9
**17**	9.53	11.14
**18**	10.66	12.4
**19**	11.01	12.9
**20**	11.79	13.63
**21**	11.18	12.9
**22**	12.18	13.94
**23**	12.1	13.9

---

[1] Hansen, P.E Isotope Effects on Nuclear Shielding. Intra-molecular Hydrogen- bonded Ketones,Aldehydes and Esters. *Magn.Reson.Chem.* **1993**, *31*, 23-37.

[2] Pittelkow, M.; Boas, U. Jessing, M.; Jensen, K. J.; Christensen**,** J. B. Role of the peri-effect in synthesis and reactivity of highly substituted naphthaldehydes: a novel backbone amide linker for solid-phase synthesis. *Org.Biol.Mol.Chem*. **2005**, *3*, 508-51.
